# Anti-epileptic drug exposure during pregnancy and neonatal birth weight outcomes: protocol for a systematic review and meta-analysis

**DOI:** 10.1186/s13643-021-01711-8

**Published:** 2021-05-29

**Authors:** Alekhya Lavu, Christine Vaccaro, Walid Shouman, Silvia Alessi Severini, Sherif Eltonsy

**Affiliations:** College of Pharmacy, University of Manitoba, 750 McDermot Ave W, Winnipeg, MB R3E 0T5 Canada

**Keywords:** Small for gestational age, Low birth weight, Epilepsy, Anti-epileptic drugs, Birth weight outcomes

## Abstract

**Background:**

The prevalence of epilepsy in pregnant women is estimated at 0.3-1%. Anti-epileptic drug (AED) exposure in-utero has been associated with various adverse health outcomes in neonates, including adverse birth weight outcomes.

**Objective:**

This review aims to summarize the published evidence on the association between AED exposure in pregnancy and adverse birth weight outcomes

**Methods:**

Studies assessing AED exposure in pregnancy and neonatal birth weight outcomes, including small for gestational age (SGA), low birth weight (LBW), birth weight (BW), length, head circumference, and cephalization index will be identified in MEDLINE®, EMBASE, Cochrane Library, Scopus, Cumulative Index of Nursing and Allied Health Literature (CINAHL), International Pharmaceutical Abstracts (IPA), and Global Health. Open grey, Theses Canada, and ProQuest Dissertations will be used to locate gray literature. Eligible study designs will include both intervention and non-interventional studies. We will not impose any time limit in the review. We will use the Newcastle-Ottawa Scale to assess the methodological quality of observational studies and quasi-experimental studies included in the review. The risk of bias of experimental studies will be appraised using the Cochrane risk-of-bias tool for randomized trials (RoB 2). A meta-analysis will be conducted using a random-effects model.

**Discussion:**

The results from this review could improve clinicians’ prescribing decisions by highlighting the safest AEDs for women who are pregnant or planning to conceive based on the evidence currently available.

**Systematic review registration:**

PROSPERO CRD42020192713

**Supplementary Information:**

The online version contains supplementary material available at 10.1186/s13643-021-01711-8.

## Background

Epilepsy is a neurological condition that affects 50 million people worldwide [1]. The prevalence of epilepsy in pregnant women is estimated at 0.3-1% [2, 3]. Anti-epileptic drug (AED) exposure in utero has been associated with various adverse health outcomes in neonates including congenital malformations, intrauterine growth restriction, neurological complications, and adverse birth weight outcomes [4–7]. Previous reports suggests that any AED exposure during pregnancy is associated with an increased risk of infants having a low birth weight (LBW) and being small for gestational age (SGA) [5, 8, 9]. Infants with adverse birth weight outcomes like SGA are at a higher risk of stillbirth, impaired thermoregulation, and hypoglycemia at birth [10]. Studies have also linked SGA with various long-term health outcomes, including impaired neurodevelopment throughout childhood, as well as cardiovascular diseases and diabetes in adulthood [11–16]. The safety profile of AEDs varies based on the molecule, generation/class, and dose used [17, 18]. AEDs have been broadly classified into old generation AEDs and new generation AEDs [19]. Old generation AEDs were the first introduced drugs to treat epilepsy during the early 1900s, whereas new generation AEDs were first used in the management of epilepsy in the 1970s [19]. Evidence on the perinatal risk of old generation AEDs are relatively established when compared to new generation AEDs [17]. First-generation AEDs have been strongly associated with SGA compared to new generation AEDs, specifically valproic acid exposure is associated with an increased risk of SGA compared to the new generation AED lamotrigine, which is believed to be safe [17, 18].

Systematic reviews summarizing the published evidence on AED’s safety in pregnancy have mainly focused on malformations and neurological outcomes [17, 20, 21]. A limited number of systematic reviews have been published on AEDs exposure during pregnancy and neonatal birth weight outcomes [22–24]. The most common limitation observed in the previously published systematic review is using fetal growth restriction (FGR) and intrauterine growth restriction (IUGR) interchangeable with SGA. SGA is a term used to describe newborns who are smaller in size than the usual for their gestational age and is defined as birth weight less than 10th percentile for the same gestational age. FGR, also known as IUGR, implies that fetal growth is being inhibited and that the fetus does not attain its growth potential [25]. Therefore, it is to say, not all SGA infants have FGR and some larger infants can still be SGA [25]. Therefore, substituting SGA for FGR might potentially affect the results of any systematic review. One recent systematic review by Chen et al. in 2017 defined FGR using SGA definition; therefore, using FGR as a substitute for SGA might have potentially excluded some of the studies which used SGA exclusively in the manuscripts [23]. Another systematic review by Viale et al. published in 2015 defined FGR as having either SGA or LBW; by doing this, they failed to differentiate the risk of an infant being born SGA and LBW separately [22] Other published reviews are at least two decades old [26]. Moreover, several original reports were published on new generation AEDs in the past 20 years; hence, the need for an up-to-date review to summarize and evaluate the risks associated with the use of AEDs in pregnancy, specifically adverse birth weight outcomes.

## Objective

This systematic review aims to summarize the evidence on the association between AEDs exposure during pregnancy and neonatal birth weight outcomes.

## Methods/design

A systematic review protocol was developed and registered in the PROSPERO database (CRD42020192713) on 19 August 2020. Preparation of our systematic review protocol was done following the guidelines of Preferred Reporting Items for Systematic Reviews and Meta-Analyses Protocols (PRISMA-P) checklist [27].

### Eligibility criteria

#### Inclusion criteria

This systematic review consists of articles that include women exposed to AEDs during pregnancy. Exposures during the first, second, and third trimesters will be considered. Polytherapy and monotherapy will also be included. No restrictions will be applied in terms of the comparison group, as we will include studies on women with epilepsy (WWE) on AEDs (active comparison), WWE not on AEDs, women without epilepsy (WWOE) on AED, and WWOE not on AED. Study designs that will be included in the review include (1) intervention studies (randomized controlled trials, quasi-randomized trials, and non-randomized trials) and (2) non-interventional studies, including observational studies (cohort studies, case-control studies, and cross-sectional studies), as well as quasi-experimental studies. Only studies published in English and French will be considered. Studies that fulfill our eligibility criteria will be included in the review (see Table 1 for PICO).

**Table 1:** Eligibility criteria to be included in the review (PICO)

**(P)opulation**	Pregnant women
**(I)ntervention**	Anti-epileptic drugs (AED) therapy
**(C)omparison**	WWOE on AED WWOE not on AED (general population) WWE not on AED WWE on AED (active comparisons)
**(O)utcome**	Primary outcomes: Birth weight outcomes (SGA, LBW, BW) Secondary outcomes: Head circumference, cephalization index, and birth length (height).

*WWOE* women without epilepsy, *WWE* women with epilepsy, *AED* anti-epileptic drug, *SGA* small for gestational age, *LBW* low birth weight, *BW* birth weight

#### Exclusion criteria

To avoid excluding studies that may have misclassified SGA as FGR and IUGR, we will include studies with IUGR or FGR as their outcome in the search strategy. After examining their definitions, studies that identified IUGR or FGR differently from SGA will be excluded during screening. Studies in which AEDs are given exclusively to infants and not to mothers during pregnancy will be excluded. Animal studies, studies containing no original research or data (e.g., reviews), conference abstracts, case reports, case series, and editorial letters will also be excluded.

### Outcome measures

Primary outcomes for this systematic review include the following:

- Small for gestational age (SGA): A birth weight classified as small for gestational age within the study, or when SGA is not named explicitly but defined as infants ≤ 10th percentile in birth weight, based on birth weight, gestational age.
- Low birth weight (LBW): A birth weight classified as low birth weight as defined within the study or birth weight less than 2500 grams (g).
- Birth weight (BW): weight at birth in grams (g).

Secondary outcomes include head circumference, cephalization index, and birth length (height) defined as:

- Head circumference: Head circumference at birth in centimeters (cm).
- Length: Height of the infant in centimeters (cm).
- Cephalization index: Ratio of the head circumference (HC) to body weight.

Studies that report any of the outcomes defined above with different terminology (e.g., fetal growth restriction or intrauterine growth restriction as a substitute for SGA) will be reclassified according to the terms specified above.

### Information sources and literature search

A systematic search strategy was developed with the assistance of a librarian. We will search MEDLINE®, EMBASE, Cochrane Library, Scopus, Cumulative Index of Nursing and Allied Health Literature (CINAHL), International Pharmaceutical Abstracts (IPA), and Global Health for relevant studies. See Additional file 1 for the search strategy that will be used in MEDLINE. The MEDLINE search strategy will be translated to other databases based on their properties. There will not be any time restrictions. Gray literature search will include Open grey, Theses Canada, and EBSCO Open Dissertations. ProQuest Dissertations will be searched using relevant keywords to locate additional studies and reports that are not published in the seven electronic databases previously stated.

The following key terms and subject headings (MeSH) will be used in various combinations and adapted according to each included database: anticonvulsants, convulsion, epilepsy, body weight, infant, low birth weight, small for gestational age, fetal growth restriction, body height, body size, cephalometry, fetal development, pregnancy outcome, pregnant woman, and prenatal exposure.

### Study selection process

Two authors (AL and CV) will independently screen titles, abstracts, and full-text articles for studies that meet our inclusion criteria using Rayyan (https://www.rayyan.ai/), a free web-tool for the screening process, as determined by our eligibility criteria [28, 29]. Any disagreements will be resolved by consensus with a third reviewer. Two authors (SE and WS) will independently screen relevant studies in French identified by AL and CV during the title and abstract screening. We also plan to hand-search the references of the final selected studies to locate additional eligible articles. If there are two publications from the same study with overlapping populations, we will choose the latest publication to be included in the review. We will use the PRISMA guidelines to report the study results.

### Data collection process

Two team members (AL and CV) will independently extract information from eligible studies using a data extraction tool. A third member will revise the chart and help resolve any disagreements. Data will include study design, country, data source, drug exposure, sample sizes in exposure and control groups, control groups definitions, birth weight outcomes, exposed and unexposed numbers (raw data) of birth weight outcomes, effect estimates including crude and adjusted odds ratios, prevalence ratios, relative risks and mean differences, and their confidence intervals and *p* values.

### Methodological quality/risk of bias appraisal

We will use the Newcastle-Ottawa Scale to assess the methodological quality of observational studies and quasi-experimental studies included in the review. The risk of bias of experimental studies will be appraised using the Cochrane risk-of-bias tool for randomized trials (RoB 2) [30]. Funnel plots and Egger’s test will be used to assess publication bias, and any probable publication bias will be assessed by the fill and trim method [31]. Kappa value will be calculated to assess the agreement between the reviewer’s screening and methodological quality scores [32]. The risk of bias analysis will be conducted by two reviewers independent of each other, and any disagreement will be resolved by consensus.

### Synthesis of included studies

Characteristics of the included studies for primary and secondary birth weight outcomes will be presented both descriptively and in tables. Pooled estimates of SGA, LBW, BW, head circumference, height, and cephalization index will be presented. Clinical and methodological heterogeneity of included studies will be examined qualitatively. Cochrane’s Q test (chi-squared) and Higgins I^2^ statistics will be used to assess the statistical heterogeneity of the included studies before conducting the meta-analysis. We will also assess the strength of the evidence and combine the results quantitatively. Whenever homogeneity is considered sufficient for an outcome, a meta-analysis will be conducted. We will conduct a random-effects meta-analysis to calculate pooled odds ratios for dichotomous data and pooled mean differences for continuous data [33]. The study weights, size of the effect, effect consistency, and direction of effect will be represented in each forest plot [33].

Funnel plots will be depicted for primary and secondary outcomes, including at least ten studies to explore asymmetry that might be explained by clinical, statistical, and methodological heterogeneity [34]. When the individual peculiarities of the studies under investigation are identified, sensitivity analysis will be done accordingly and reported in figures [33]. With a sufficient number of studies, subgroup analysis will be conducted by dose, AED exposure period during pregnancy (first trimester, second trimester, and third trimester), and therapy type (monotherapy and polytherapy) and will be reported in figures.

## Discussion

It is essential to understand how AEDs exposure in pregnancy affects birth weight outcomes in offspring. This is particularly important due to the increased prescribing rates of new-generation AEDs, and the limited evidence of their fetal safety. Cautious prescribing practices of AEDs in women of reproductive age are crucial, as more than 30% of pregnancies are not planned, and some AEDs can potentially reduce contraceptives’ efficacy [35, 36]. Therefore, placing women of childbearing age on AEDs should be based on recommended care, with sufficient evidence on neonates’ safety. Clinicians must make medication choices by balancing the risk of increased seizure frequency in mothers versus the potential of adverse fetal outcomes caused by AED exposure [37].

There is a need for a well-constructed systematic review focusing on an array of adverse birth weight outcomes potentially caused by AED exposure during pregnancy. We anticipate the presence of fewer published reports on new generation AEDs and new versus old generation AEDs. This review will locate reports from numerous sources including gray literature and will summarize, pool, and evaluate the methodological quality of the studies in a systematic approach.

This will help us expand the knowledge on how the safety of AED during pregnancy varies based on the type of AED and the combination of prescribed AEDs. It will also help physicians make a careful decision regarding the safety of AED use in pregnancy and choose the safest treatment regimen. The knowledge generated through this project will also help in the pre-counseling of women who plan to be pregnant and will help them make rational evidence-based choices along with their physicians.

## Supplementary Information

**Additional file 1.** MEDLINE searchR2

**Additional file 2.** PRISMA-P checklistR2

**Additional file 3.** list of AEDsR2

**Additional file 4.** Screening questions_SER2

## Data Availability

The datasets during and/or analyzed during the current study are available from the corresponding author on reasonable request.

## Abbreviations

AEDs: Anti-epileptic drugs
SGA: Small for gestational age
LBW: Low birth weight
BW: Birth weight
WWE: Women with epilepsy
WWOE: Women without epilepsy
IUGR: Intrauterine growth restriction
FGA: Fetal growth restriction
IPA: International Pharmaceutical Abstracts
CINAHL: Cumulative index of nursing and allied health literature
RoB 2: Cochrane risk-of-bias tool for randomized trials
PRISMA-P: Preferred Reporting Items for Systematic Reviews and Meta-Analyses Protocols
HC: Head circumference
