# Supplementary material for: Anti-epileptic drug exposure during pregnancy and neonatal birth weight outcomes: protocol for a systematic review and meta-analysis
Source: Syst Rev. 2021 May 29;10:159. doi: 10.1186/s13643-021-01711-8 (PMC8164239; doi:10.1186/s13643-021-01711-8)
Supplement: Supplementary file 1 — Additional file 1. MEDLINE searchR2 [file 13643_2021_1711_MOESM1_ESM.docx]

| **#** | **Searches** |
| --- | --- |
| 1. 14 | Exp anticonvulsants/ |
|  | (agent* or drug* adj2 (anticonvuls* or anti convuls* or antiepilep* or anti epilep*)).tw,kw. |
|  | (acetazolamide or bromides or carbamazepine or chlormethiazole or clobazam or clorazepate dipotassium or diazepam or dimethadione or estazolam or ethosuximide or felbamate or flunarizine or gabapentin or lacosamide or lamotrigine or levetiracetam or lorazepam or magnesium sulfate or medazepam or mephenytoin mephobarbital or meprobamate or nitrazepam oxcarbazepine or paraldehyde or phenobarbital or phenytoin or pregabalin or primidone or riluzole or thiopental or tiagabine or tiletamine or topiramate or trimethadione or valproic acid or vigabatrin or zonisamide).tw,kw. |
|  | Exp seizures/ dt |
|  | Exp epilepsy/ dt |
|  | (convulsion or seizure or epilepsy).tw,kw. |
| 1. 19 | Brivaracetam/ |
| 1. 20 | (brivaracetam or brivlera or briviact).tw,kw. |
| 1. 23 | Eslicarbazepine/ |
| 1. 24 | (eslicarbazepine* or apitom or zebinix).tw,kw. |
| 1. 25 | Fosphenytoin sodium/ |
| 1. 26 | (fosphenytoin* or cerebyx or pro-epanutin).tw,kw. |
| 1. 27 | Ganaxolone/ |
| 1. 28 | Ganaxolone.tw,kw. |
| 1. 31 | Losigamone/ |
| 1. 32 | Losigamone.tw,kw. |
| 1. 33 | Perampanel/ |
| 1. 34 | (perampanel or fycompa).tw,kw. |
| 1. 35 | Piracetam/ |
| 1. 36 | Piracetam.tw,kw. |
|  | Pregabalin/ |
| 1. 38 | (pregabalin or lyrica or lecaent or rewisca).tw,kw. |
| 1. 39 | Remacemide/ |
| 1. 40 | (remacemide*).tw,kw. |
| 1. 41 | Retigabine/ |
| 1. 42 | (retigabine or ezogabine or trobalt).tw,kw. |
| 1. 43 | Rufinamide/ |
| 1. 44 | (rufinamdie or banzel or inovelon).tw,kw. |
| 1. 45 | Safinamide/ |
| 1. 46 | (safinamide or xadago).tw,kw. |
| 1. 47 | Stiripentol/ |
| 1. 48 | (stiripentol or diacomit).tw,kw. |
| 1. 53 | Or/1-32**[anticonvulsants concept]** |
| 1. 54 | Exp body weight/ |
|  | Body weight*.tw,kw. |
|  | Birth weight/ |
|  | Birth weight*.tw,kw. |
|  | Fetal weight/ |
|  | (fetal or foetal) adj weight*.tw,kw. |
|  | Exp infant, low birth weight/ |
|  | Sga, small for gestational age.tw,kw. |
|  | (low adj2 (birthweight* or birth weight*) or lbw*).tw,kw. |
|  | Fetal growth retardation/ |
|  | ((fetal or foetal or intrauterine) adj (growth retard* or growth restrict*)).tw,kw. |
|  | Body height/ |
|  | Body height.tw,kw. |
|  | Body size/ |
|  | (body adj2 size*).tw,kw. |
|  | Cephalometry/ |
|  | (cephalometry or craniometry).tw,kw. |
|  | Head/ |
|  | Head.tw,kw. |
|  | Fetal development/ |
|  | ((fetal or foetal) adj (develop* or growth)).tw,kw. |
|  | Pregnancy outcome/ |
|  | (pregnancy outcome).tw,kw. |
|  | Or/34-56 [birth size concept] |
|  | Exp pregnancy/ |
|  | Pregnant woman/ |
|  | Infant, newborn/ |
|  | (infant* or newborn*).tw,kw. |
|  | Gestat*.tw,kw. |
|  | Pregnan*.tw,kw. |
|  | (prenatal* or pre natal*).tw,kw. |
|  | Prenatal exposure/ |
| 1. 75 | Or/58-65 [pregnancy concept] |
| 1. 76 | 33 and 57 and 66 |
| 1. 78 | Limit 67 to (english or french) |
|  | \| Exp animals/ not (exp animals/ and humans/) \|  \| \| --- \| --- \| |
| 1. 82 | 68 not 69 |
