# Supplementary material for: Anti-epileptic drug exposure during pregnancy and neonatal birth weight outcomes: protocol for a systematic review and meta-analysis
Source: Syst Rev. 2021 May 29;10:159. doi: 10.1186/s13643-021-01711-8 (PMC8164239; doi:10.1186/s13643-021-01711-8)
Supplement: Supplementary file 3 — Additional file 3. list of AEDsR2 [file 13643_2021_1711_MOESM3_ESM.docx]

**LIST OF AEDS INCLUDED IN THE STUDY**

|  | Generic Name |
| --- | --- |
| **First Generation** | |
| 1 | Carbamazepine |
| 2 | Diazepam |
| 3 | Phenytoin |
| 4 | Valproic acid |
| 5 | Ethosuximide |
| 6 | Clonazepam |
| 7 | Phenobarbital |
| 8 | Bromides |
| 9 | Clomethiazole |
| 10 | Clorazepate dipotassium |
| 11 | Paraldehyde |
| 12 | Thiopental |
| **Second Generation** | |
| 1 | Lamotrigine |
| 2 | Gabapentin |
| 3 | Pregabalin |
| 4 | Oxcarbazepine |
| 5 | Levetiracetam |
| 6 | Topiramate |
| 7 | Tiagabine |
| 8 | Lacosamide |
| 9 | Rufinamide |
| 11 | Vigabatrin |
| 12 | Clonazepam |
| 13 | Clobazam |
| 14 | Lorazepam |
| 15 | Primidone |
| 16 | Felbamate |
| 17 | Zonisamide |
| 18 | Acetazolamide |
| 19 | Estazolam |
| 20 | Flunarizine |
| **Third Generation** | |
| 1 | Perampanel |
| 2 | Brivaracetam |
| 3 | Safinamide |
| 4 | Stiripentol |
| 5 | Eslicarbazepine |
| 6 | Felbamate |
| 7 | Fosphenytoin |
| 8 | Losigamone |
| 9 | Retigabine |
| 10 | Safinamide |
| 11 | Stiripentol |
| 12 | Ganaxolone |
| 13 | Remacemide |
