# Supplementary material for: Anti-epileptic drug exposure during pregnancy and neonatal birth weight outcomes: protocol for a systematic review and meta-analysis
Source: Syst Rev. 2021 May 29;10:159. doi: 10.1186/s13643-021-01711-8 (PMC8164239; doi:10.1186/s13643-021-01711-8)
Supplement: Supplementary file 4 — Additional file 4. Screening questions_SER2 [file 13643_2021_1711_MOESM4_ESM.docx]

**Screening steps for located studies**

Level 1 Screening (Titles)

1. Was the study conducted exclusively in humans?

YES____ NO____ UNCLEAR____

1. Does the study include pregnant women?

YES____ NO____ UNCLEAR____

1. Is this a relevant study design (experimental studies, quasi-experimental studies, observational studies)?

YES____ NO____ UNCLEAR____

1. Does the study report perinatal/neonatal outcomes in the newborn?

YES____ NO____ UNCLEAR____

Level 2 Screening (Abstract)

1. Does the study include pregnant women?

YES____ NO____ UNCLEAR____

1. Does the study include pregnant women with epilepsy or exposed to AED*?*

YES____ NO____ UNCLEAR____

1. Does the study report at least one outcome of interest (small for gestational age (SGA), low birth weight (LBW), birth weight (BW), head circumference, length/height, cephalization index)?

YES____ NO____ UNCLEAR____

1. Is this a relevant study design (experimental studies, quasi-experimental studies, observational studies)?

YES____ NO____ UNCLEAR____

Level 3 Screening (Full text)

1. Does the study include pregnant women exposed to any of the AEDs predefined (monotherapy or combination)*?*
   1. YES____ NO____
2. Does the study report at least one outcome of interest (small for gestational age (SGA), low birth weight (LBW), birth weight (BW), head circumference, length/height, cephalization index)?
   1. YES____ NO____
3. Is this a relevant study design (experimental studies, quasi-experimental studies, observational studies)?
   1. YES____ NO____
